# Clinical and misdiagnosed analysis of primary pulmonary lymphoma: a retrospective study

**DOI:** 10.1186/s12885-018-4184-1

**Published:** 2018-03-12

**Authors:** D. Yao, L. Zhang, P. L. Wu, X. L. Gu, Y. F. Chen, L. X. Wang, X. Y. Huang

**Affiliations:** 1 The First Affiliated Hospital of Wenzhou Medical University & Key Laboratory of Heart and Lung, Zhejiang, 325035 China; 2 Wenzhou Medical University, Zhejiang, 325035 China

**Keywords:** Primary pulmonary lymphoma, Misdiagnosis, Pathology, Biopsy

## Abstract

**Background:**

The primary pulmonary lymphoma (PPL), with a low incidence, was highly misdiagnosed in clinic. The present study analyzes the clinical features, laboratory and imaging data, pathologic characteristics, and summarizes misdiagnosis reasons of PPL cases, aims to provide a better understanding and increase the accuracy of early diagnosis and minimize the misdiagnosis of PPL.

**Methods:**

The clinical data of 19 cases were collected from the first affiliated hospital of Wenzhou medical university (PRC) from April 2010 to May 2016. All cases were confirmed by pathology. The process of misdiagnosis was described. This study retrospectively analyzed the incidence, clinical presentation, laboratory examination, Chest CT scan and diagnosis of the cases.

**Results:**

The symptoms of the 19 cases were dyspnea, fever, hemoptysis, chest pain or physical findings without obvious symptoms. Five patients were pneumonia-like, nine patients had lung single nodule or mass and four patients got pleural effusion, which were reported by computed tomography (HRCT) scan. There were 2 cases of Hodgkin lymphoma (HL), and 17 cases of non-Hodgkin lymphoma (NHL). In NHL cases, 12 cases were confirmed mucosa associated lymphoid tissue B lymphoma type, 3 cases were confirmed diffuse large B-cell lymphoma, angioimmunoblastic T-cell lymphoma and ALK positive anaplastic large cell lymphoma were one case separately. Clinical and imaging manifestation of PPL is untypical, but there are still some hints: 1) Fuzzy shadow at the edge of lung mass with air bronchogram; 2) Lung mass shadow stable for a long time; 3) Pneumonia-like changing without infections clinical and lab manifestation. Thirteen patients (68.4%) were misdiagnosed as pneumonia, lung cancer and tuberculosis initially. The term between initial diagnosis and final diagnosis lasted for half a month up to 2 years, with median time of 6 months. Two cases were misdiagnosed as tuberculosis. One case was misdiagnosed as small cell lung cancer.

**Conclusion:**

Clinical and imaging manifestation of PPL is untypical. Biopsies should be taken actively if the imaging findings don’t match the symptoms or the anti-infection treatments to “lung infection” don’t work. Accurate diagnosis requires adequate tissue sampling with appropriate ancillary pathologic studies. If clinical manifestation and the diagnosis don’t match, repeated biopsy should be ordered.

## Background

Primary pulmonary lymphoma (PPL), as an uncommon disease, affects the lung occuring over a broad clinical and pathologic spectrum. PPL, with a low incidence, comprises < 1% [1] of all non-Hodgkin’s lymphoma (NHL), and 0.5% of all primary pulmonary malignant tumors [2, 3]. Traditionally, it has been defined as a clonal lymphoid proliferation affecting one or both lungs (parenchyma and/or bronchi) in a patient with no clinical, pathological, or radiographic evidence of lymphoma elsewhere, either in the past, or at present, or for 3 months after presentation [4, 5]. The vast majority are type of low-grade mucosa-associated lymphoid tissue [6–10]. Although there have been several retrospective reports, few studies were investigated in the Asian population [8–10].

PPL usually occurs with nonspecific clinical features, is highly misdiagnosed in clinic. In order to provide a better understanding of this disease, increase the accuracy of early diagnosis and minimize misdiagnosis, this research was to analyze and summarize the misdiagnosis reasons of PPL based on the clinical features, the laboratory and imaging examinations, and the pathologic characteristics of PPL cases from our hospital during April 2010 to May 2016 while referring to relative literature.

## Methods

The clinical data of 19 cases of histologically proven PPL were collected from the first affiliated hospital of Wenzhou Medical University from April 2010 to May 2016. At the same time, there were 760 cases of lymphoma collected in the hospital. All the 19 cases were confirmed by pathology. Medical records about clinical characteristic, the time of diagnosis, treatment and follow-up were systematically reviewed, including clinical features, laboratory findings, radiographs, bronchoscopic features and pathological data. Ethical approval for this investigation was obtained from the Research Ethics Committee, the First Affiliated Hospital of Wenzhou Medical University. And all patients consent to participate and all data were consent for publish.

## Results

### Clinical manifestation and laboratory examination

Within the 19 cases, 11 male and eight female patients were described with a median age of 51.8 years old (range 19 to 71 years). The symptoms (Table 1) of the 19 cases were dyspnea, fever, hemoptysis, chest pain or physical findings with no obvious symptoms. LDH exam showed 4 cases elevation, and β2 microglobulin exam showed 2 elevation in these 19 patients.

**Table 1:** Clinical and laboratory features of patients with PPL

Features	Cases	Percent%
Age		
< =60	13	68.4
> 60	6	31.6
Sex		
male	11	57.9
female	8	42.1
No symptoms	6	31.6
Respiratory symptom		
cough,	10	52.6
dyspnea	4	21.1
fever	3	15.8
hemoptysis	2	10.5
chest pain	4	21.1
β2 microglobulin rise	2	10.5
LDH rise	4	21.1

### Chest CT scan

**Fig. 1:**
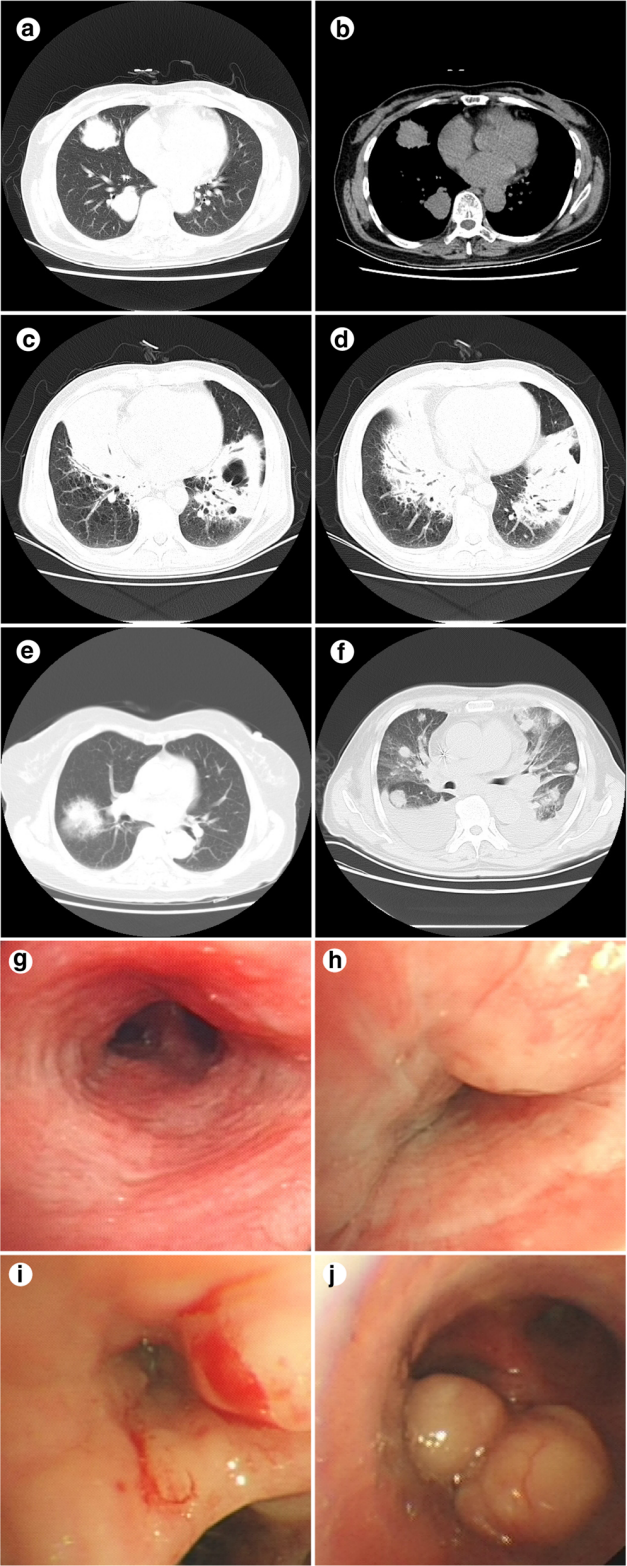
**a**-**f**:chest CT scan (**a**-**b**: multiple nodules, **c**-**d**:pneumonia-like, e:lung single nodule or mass and pleural effusion, **f**: multiple nodules with pleural effusion); **g**-**j**: bronchscopy (**g**:mucosal hyperemia and edema, **i**-**j**: tumor growth structure, **h**: tracheal narrow with external pressure)

High resolution computed tomography (HRCT) of the chest (Table 2) revealed unilateral disease in 13 patients (Fig. 1a-b: showed multiple nodules), bilateral disease in six patients, pneumonia-like (Fig. 1c-d) in five patients, lung single nodule or mass (Fig. 1e) in nine patients and pleural effusion (Fig. 1f: showed multiple nodules with pleural effusion) in four patients. None of these 19 patients accompanied with mediastinal, axillary or cervical lymph node enlargement (Fig. 1a to f).

**Table 2:** High resolution computed tomography (HRCT) features

Features	Cases	Percent%
bilateral disease	6	31.6
unilateral disease	13	68.4
Single nodule or mass	9	47.4
Multiple nodules	1	5.26
Pneumonia-like	4	21.1
Mixed type	5	26.3
Pneumonia-like with cavity	1	5.26
Pneumonia-like with pleural effusion	1	5.26
Nodules with pleural effusion	3	15.8

### Bronchoscopy

Four patients were confirmed by flexible bronchoscopy. Mucosal hyperemia and edema (Fig. 1g) were found in one patient, tumor growth structure (Fig. 1i-j) was found in two patients, tracheal narrow with external pressure (Fig. 1h) was found in one patient. Normal were reported in 15 patients.

### Pathologic characteristics

Pathologic characteristics were performed by pathologist in all 19 cases (Table 3). The lung structure was replaced by tumor cells. Two Hodgkin lymphoma (HL) cases were confirmed to the type of tuberous sclerosis, and 17 non-Hodgkin lymphoma (NHL) cases were confirmed by histopathology. Among 17 NHL cases, 12 cases were confirmed mucosa associated lymphoid tissue B lymphoma type, 3 cases were confirmed diffuse large B- cell lymphoma, one case were confirmed angioimmunoblastic T-cell lymphoma and ALK positive anaplastic large cell lymphoma separately.

**Table 3:** Pathologic types of the 19 cases

Pathologic type	Cases	Percent%
HL	2	10.5
MALT	12	63.2
Diffuse large B-cell lymphoma	3	15.8
Angioimmunoblastic T-cell lymphoma	1	5.26
ALK (+) anaplastic large cell lymphoma	1	5.26

### Diagnostic procedures and prognosis

Thirteen patients (68.4%) were misdiagnosed as pneumonia, lung cancer, and tuberculosis before confirming, and accepted anti-infection, anti- tuberculosis or anti-tumor treatment. The time from initial diagnosis to the final diagnosis processed from half month up to 2 years, with the medians time of 6 months. Ten patients were receiving antibiotic treatment for presumptive non-resolving pneumonia. Two cases were misdiagnosed as tuberculosis, one has no obvious symptoms but was reported has multiple nodules by CT scan, and accepted anti-tuberculosis treatment for more than 1 year. But during the anti- tuberculosis treatment the lesions processed cavity and then the patient accepted CT-guided percutaneous lung biopsies subsequently, at last PPL was confirmed by histopathology. Another one, whose chief complain was cough, with the imaging of pneumonia-like and T-SPOT positive, accepted anti-tuberculosis treatment for 1 month and ceased voluntarily. So he went back to hospital and accepted biopsy while cough symptoms processed. Another case was misdiagnosed as small cell lung cancer whose bronchoscope found right bronchial neoplasm. Pathology of the biopsy tissue presented poorly differentiated carcinoma, combined with immunohistochemical presented neuroendocrine differentiation, was misdiagnosed as small cell lung cancer treated with EP (etoposide + carboplatin) chemotherapy subsequently. Four monthes later, the patient accepted bronchoscopy biopsy once again for sustained fever, and was confirmed ALK positive anaplastic large cell lymphoma finally (Tables 4 and 5).

**Table 4:** Misdiagnosed cases

Initial diagnosis	Cases	Chief complaint	Time of misdiagnosis(months)	Pathological diagnosis obtained
pneumonia	10	None, cough, dyspnea or chest pain	0.5–24	CT-guided percutaneous lung biopsy, bronchoscopy, surgery and thoracoscopy
tuberculosis	2	Cough, none	1–12	CT-guided percutaneous lung biopsy
lung cancer	1	Cough and fever	4	bronchoscopy

**Table 5:** Treatment of 13 misdiagnosed cases

Case No.	Initial diagnosis	Chief complaint	CT scan	Treatment
1	Pneumonia	None	Pneumonia-like	anti-infection treatment with levofloxacin for half a month
2	Pneumonia	None	Nodules with pleural effusion	anti-infection treatment with cefperazone-sulbactam for half a month
3	Pneumonia	Cough	Mass	anti-infection treatment with cefperazone-sulbactam for a month
4	Pneumonia	Chest pain	Pneumonia-like	anti-infection treatment with ceftriaxone sodium and levofloxacin irregularly in local hospital, 8 months later he came to our hospital and accepted biopsy
5	Pneumonia	Cough	Pneumonia-like	anti-infection treatment with ceftriaxone sodium for half a month, 6 months later he came back to hospital
6	Pneumonia	Chest pain, dyspea and fever	Pleural effusion	anti-infection treatment with ceftriaxone sodium for half a month
7	Pneumonia	None	Mass	anti-infection treatment with levofloxacin for half a month
8	Pneumonia	Cough, dyspea and fever	Mass	anti-infection treatment with cefperazone-sulbactam for half a month
9	Pneumonia	Cough	Mass	anti-infection treatment with levofloxacin for a month
10	Pneumonia	Cough and chest pain	Mass	anti-infection treatment with ceftriaxone sodium for half a month, 3 months later he came back to hospital
11	Tuberculosis	Cough	Pneumonia-like	anti-tuberculosis treatment for 1 month and ceased voluntarily
12	Tuberculosis	None	Multiple nodules	anti-tuberculosis treatment for more than 1 year
13	Lung cancer	Cough and fever	Multiple nodules	treated with EP (etoposide + carboplatin) chemotherapy

Pathological diagnosis was obtained by CT-guided percutaneous lung biopsies in ten patients, while four patients were confirmed by flexible fiberoptic bronchoscopy, 4 cases were confirmed by surgery, and the other one with pleural effusion, were confirmed by biopsies through thoracoscopy.

Prognosis of PPL was generally good, with treatment options ranging from surgical resection in localized cases to chemotherapy in more diffuse involvement. Nineteen patients received various combinations of treatment, surgery plus chemotherapy in four patients, and chemotherapy alone in 15 patients. No patient was treated with radiotherapy. The regimes of chemotherapy were cyclophosphamide, adriamycin, vincristine and prednisone (CHOP), cyclophosphamide, vincristine and prednisone (CVP), rituximab plus CHOP (R-CHOP) and rituximab plus CVP (R-CVP).

## Discussion

PPL [11, 12], as an extremely rare disease, represented less than 1% of lung malignancies, and less than 1% of non-Hodgkin’s lymphoma (NHL) and accounting for only 3.6% of extranodal lymphomas. There were almost one third of patients (31.6%) with no symptoms in our research. PPL, with a low incidence, usually occured with nonspecific clinical features, and was highly misdiagnosed in clinic. In our research, 13 patients (68.4%) were misdiagnosed as pneumonia, lung cancer, tuberculosis before confirming. The present study analyzes the clinical features, laboratory and imaging data, pathologic characteristics, and summarize misdiagnosis reasons of PPL cases. Aims to provide a better understanding and increase the accuracy of early diagnosis and minimize the misdiagnosis of PPL.

The symptoms and physical signs of pulmonary lymphoma were highly heterogeneous. In our research, 11 male and eight female patients were described with a mean age of 51.8 years old (range 19 to 71 years). It occured most commonly around the age of 50–70 years, although rare cases of younger age of onset had been reported. LDH exam showed 4 cases (21%) elevation, and β2 microglobulin exam showed 2 (10.5%) elevation in these 19 patients. Laboratory tests showed no-specific.

Low grade B-cell PPL, as most commonly in PPL, formed almost 85–90% of all reported cases, out of which almost all cases correspond to MALT-type NHL [13–15]. The recent delineation of new pathologic entities such as low-grade malignant lymphoma of mucosa-associated lymphoid tissue (MALT type) had aided in the understanding of the pathophysiology, clinical course, and management of patients with pulmonary lymphoma. Ahmed [16] reported no clinical symptoms in the MALT lymphoma diagnosis was about 40.9%. While 63.2% (12/19) MALT lymphoma were found in our study. The pathology of the six cases with no symptoms were confirmed MALT-type in our study. Nearly half of reported cases of MALT-type had been asymptomatic at presentation, while in the others, the presentation had been associated with non specific respiratory symptoms.

The radiographic presentation of PPL was not specific, but variable. The same as Bae et al.’s reported [17], single or multiple nodules or areas of consolidation were the main patterns of radiographic abnormalities in our study, accounted for 47.4%. We found that 26.3%of patients presented pneumonia-like, with air space consolidation with or without air bronchogram. Other [16–18] radiographic presentation of PPL were pleural effusion, mixed-type, multiple nodules etc. In previous case reports, PPL may appear as a single nodule, as multiple nodules or as cavitated lesions [6]. Cavitated lesions had a wide differential diagnosis including granulomatous diseases (Wegener’s granulomatosis), tuberculosis, necrotic pneumonia, pulmonary Gram-negative organisms, anaerobic bacteria or fungal infections, pulmonary abscess, evacuated hydatid cysts, septic emboli, inhalational diseases (eg, coal workers’ pneumoconiosis and silicosis), eosinophilic pneumonia, cavitated rheumatoid nodules and neoplastic diseases (primary bronchial carcinoma). Metastatic lung nodules may especially cavitate squamous cell types but also adenocarcinomas, sarcomas, melanomas and osteosarcomas [7]. Because of the wide differential diagnosis, multiple invasive and noninvasive diagnostic investigations were common. Due to these circumstances, in many cases, the invasive biopsy that confirmed the diagnosis was delayed [8].

As the clinical features of PPL were poorly defined, most of the patients were initially misdiagnosed as pneumonia, pulmonary tuberculosis, organizing pneumonia and lung cancer. Altough imaging manifestation of PPL had a less specificity [19–22], there were still some hints: 1) Fuzzy shadow at the edge of lung mass with air bronchogram; 2) Lung mass shadow stable for a long time; 3) Pneumonia-like changing without infections clinical and lab manifestation.

Although there had been several retrospective case reports, few studies were investigated in the Asian population. PPLwas commonly misdiagnosed as pneumonia, pulmonary abscess and carcinoma as reported [9, 10, 23–25]. The main cause of misdiagnosis were summarized as follow: 1) The limitation of specialists. All the patients went to the repiratory department because of symptoms or physical findings in lung or air way, some of them accepted anti-infection treatment primarily combined with symptoms, laboratory examination and CT scan. 2) Poor compliance. Some patients didn’t come back for subsequent visit when they felt a little better after anti-infection treatment, and some patients went to other hospitals while the symptoms progressed. 3) Some patients’ chest CT scan showed pneumonia-like or cavity and the report of T-SPOT was positive, but the evidence of infection were negative, given the highly endemic nature of Mycobacterium tuberculosis in China and the fact of chest CT scan, they accepted diagnostic anti-tuberculosis treatment. 4) The similarity of pathological manifestation. In some cases the pathologists were confused because similarity of pathological manifestation of PPL. In our experience, if a patient was elderly and not responsive to treatment, alternative diagnosis should be considered and invasive procedures, such as bronchoscopy, CT-guided percutaneous lung biopsies, and an open thoracotomy or a video-assisted thoracoscopic (VATS) lung biopsy should be performed.

As a consequence, PPL could be diagnosed only by pathologic methods. Previously, the pathological tissue was mainly obtained through the diseased lung surgery, open thoracotomy, under thoracoscopy biopsy. CT guided percutaneous lung biopsy, thoracotomy and flexible fiberoptic bronchoscopy were the common methods in current. In our stury, pathological diagnosis was obtained by CT-guided percutaneous lung biopsies in ten patients, while four patients were confirmed by flexible fiberoptic bronchoscopy, 4 cases were confirmed by surgery, and the other one clinical feature was pleural effusion, confirmed by thoracoscopy. Of the 19 cases, more than half of the patients’ pathology was confirmed by CT guided percutaneous lung biopsy. CT guided percutaneous lung biopsy should be taken actively if the imaging findings did not match the symptoms or the anti-infection treatments to “lung infection” didn’t work. Accurate diagnosis requires adequate tissue sampling with appropriate ancillary pathologic studies.

The therapy of PPL relies on histology [26, 27]. Surgery, chemotherapy and radiotherapy were the main treatment to PPL, alone or in combination, as well as therapeutic abstention or follow-up alone, had been commonly used. For MALT lymphoma the final results of IELSG-19 have recently been reported suggesting that rituximab+ chlorambucil should probably be the first line of chemotherapy.

## Conclusion

Clinical and imaging manifestation of PPL is untypical, the symptoms of PPL are mild, systemic symptoms are rare, and about one third of patients may have no symptoms. Imaging manifestation of PPL have a less specificity, but there are still some hints. Biopsies should be taken actively if the imaging findings did not match the symptoms or the anti-infection treatments to “lung infection” didn’t work. Accurate diagnosis requires adequate tissue sampling with appropriate ancillary pathologic studies. If clinical manifestation and the diagnosis didn’t match, repeated biopsy should be ordered.

## Abbreviations

CHOP: Cyclophosphamide, adriamycin, vincristine and prednisone
CVP: Cyclophosphamide, vincristine and prednisone
EP: Etoposide + carboplatin
HL: Hodgkin lymphoma
HRCT: Computed tomography
MALT: Mucosa-associated lymphoid tissue
NHL: Non-Hodgkin lymphoma
PPL: Primary pulmonary lymphoma
R-CVP: Rituximab plus CHOP (R-CHOP) and rituximab plus CVP
